# TGF-β superfamily members from the helminth *Fasciola hepatica* show intrinsic effects on viability and development

**DOI:** 10.1186/s13567-015-0167-2

**Published:** 2015-03-11

**Authors:** Ornampai Japa, Jane E Hodgkinson, Richard D Emes, Robin J Flynn

**Affiliations:** School of Veterinary Medicine and Science, University of Nottingham, Sutton Bonington, Nottingham, LE12 5RD, UK; Advanced Data Analysis Centre, University of Nottingham, Sutton Bonington, Nottingham, LE12 5RD, UK; Department of Infection Biology, Institute of Infection and Global Health, University of Liverpool, Liverpool, L3 5RF, UK

## Abstract

The helminth *Fasciola hepatica* causes fasciolosis throughout the world, a major disease of livestock and an emerging zoonotic disease in humans. Sustainable control mechanisms such as vaccination are urgently required. To discover potential vaccine targets we undertook a genome screen to identify members of the transforming growth factor (TGF) family of proteins. Herein we describe the discovery of three ligands belonging to this superfamily and the cloning and characterisation of an activin/TGF like molecule we term FhTLM. FhTLM has a limited expression pattern both temporally across the parasite stages but also spatially within the worm. Furthermore, a recombinant form of this protein is able to enhance the rate (or magnitude) of multiple developmental processes of the parasite indicating a conserved role for this protein superfamily in the developmental biology of a major trematode parasite. Our study demonstrates for the first time the existence of this protein superfamily within *F. hepatica* and assigns a function to one of the three identified ligands. Moreover further exploration of this superfamily may yield future targets for diagnostic or vaccination purposes due to its stage restricted expression and functional role.

**Electronic supplementary material:**

The online version of this article (doi:10.1186/s13567-015-0167-2) contains supplementary material, which is available to authorized users.

## Introduction

Transforming growth factor (TGF)–β1 is a multifaceted cytokine belonging to the TGF-β superfamily of proteins composed of the TGF and bone morphogenic proteins (BMP) subfamilies. Structurally TGF subfamily members are characterised by the presence of 9 cysteine residues while 7 residues are found in BMP proteins. TGF-β1 signalling is involved in the development and differentiation of animal tissues and organs [1,2] and its pathway components are very well conserved and seemingly evolved early in the history of animals [3]. Members of the TGF superfamily have been described in both higher and lower animals. Despite their pivotal role in animal development there is no apparent correlation regarding the complexity of morphology and the number of the signalling pathway members present [4]. Regardless, this protein superfamily has enormous diversity and specificity of signalling is defined through a combination of receptors and cognate intracellular signalling components. Upon ligation, TGF-β serine threonine kinase (STK) receptors directly activate the relevant receptor-activated Smad (R-Smad) signalling component. Smad2 and 3 transduce the TGF-β/activin signal and while Smad1, 5 and 8 mediate the BMP signal. Smad4 is subsequently joined to the activated R-Smad complex and this migrates into the nucleus to regulate expression of target genes [5].

In the model organism *Caenorhabditis elegans*, TGF-β-like ligands, Decapentaplegic BMP-like (DBL)-1 and Dauer formation abnormal (DAF)-7 have been well characterized [6]. Ce-DBL-1, a homolog of BMPs, was found to influence body size and tail formation of male worms. Mutant worms lacking DBL-1 have been shown to be smaller than wild type worms while reintroduction and overexpression of a DBL-1 expressing plasmid was found to result in longer worms [7]. Signalling by the TGF-β subfamily member DAF-7, was shown to be involved in the control of the dauer stage and mutant DAF-7 worms were found to be more susceptible to temperature change. Mutant DAF-7 worms have also been shown to enter into the arrest stage under inappropriate conditions or fail to enter the dauer stage entirely [8].

Several studies have examined the expression and localization of TGF-β homologues during the life cycle of parasitic helminths and homologues of DAF-7 have been extensively investigated amongst parasitic nematodes. It has been suggested that this ligand controls arrested development of parasitic nematodes particularly during the infective L3 stage [9]. A wide array of intestinal nematodes exhibit a high level of expression of DAF-7 homologue in their L3 stages including *Ancylostoma caninum*, [10,11], *Strongyloides ratti*, *Parastrongyloides trichosuri* [12,13], *Heligmosomoides polygyrus* and *Teladorsagia circumcincta* [14].

Within trematodes, TGF-β proteins have only been extensively studied in *Schistosoma* spp., where expression of TGF-β or BMP family members has been detected throughout the life cycle [15-17]. SmInAct is a TGF-β/Activin-like ligand, expressed in female *S. mansoni* worms and their eggs. Reduction in levels of SmInAct by RNAi resulted in stunting of the female worms and incomplete development of eggs. SmInAct was localised to embryonated eggs and female reproductive organs supporting its role in worm development [15]. Homologues of BMP proteins have also been demonstrated in *S. mansoni* (SmBMP) and *S. japonicum* (SjBMP), with levels of SjBMP expression were greatest in early larval stages and eggs of *S. japonicum* [17]. The transcript was localized to the tegument and epithelium of adults; furthermore it was also present in the ovary of the female worm. RNAi knockdown resulted in a phenotype with low egg output and stunted egg development.

*Fasciola hepatica* is a major trematode parasite of livestock and an emerging human zoonotic disease found throughout the world. *F. hepatica* completes its lifecycle through the utilisation of a mud-snail intermediate host before reaching maturity, as a hermaphrodite, within the liver and bile ducts of ruminants. Control is centred on chemotherapy but mounting drug resistance and shifting patterns of disease underline the need for novel and sustainable strategies for control. In order to develop effective novel therapeutic targets, it is important to understand parasite biology and immune evasion mechanisms. Genes previously identified as encoding for homologues of the TGF-β family are present in a number of parasitic worms and these molecules may offer novel therapeutic approaches for the control of multiple species of veterinary and medical importance, e.g. *A. caninum, S. stercoralis* and *H. contortus* [18]. Herein, we sought to identify any gene(s) encoding TGF-β homologues present in the *F. hepatica* genome given that any TGF-β molecules present may control parasite development, thus presenting an attractive target for parasite control or diagnosis.

## Materials and methods

### Genome analysis

Genome analysis was conducted using the putative *F. hepatica* genome produced in the laboratory of Dr Jane Hodgkinson University of Liverpool [19]. TGF-β like sequences were identified in the *F. hepatica* genome through a tBlastn search of the draft genome contigs using protein sequences of TGF-β1-3 from mammalian hosts and SmInAct, a TGF-β like protein from *S. mansoni* as queries. All translated potential matching regions were compared to both the non-redundant (nr) database and a database of translated bovine genes using the Blastp algorithm. An E-value cut off of 1 × 10^−4^ was used to define a significant hit. Candidate genes were identified from genomic DNA using the exonerate software (scripts available at [20]. The deduced amino acid sequences of TGF-β like proteins of *F. hepatica* and other helminths identified from PSI Blast (Position Specific Iterated Blast) and with mammalian TGF-β and BMP subfamily were aligned using MUSCLE [21] implemented in the Seaview program [22,23].

### RACE cloning, recombinant expression vector construction and protein production

First strand cDNA from adult *F. hepatica* was prepared as template for RACE using GeneRacer™ Kit (Invitrogen, UK) following the manufacturers’ protocol. 5' and 3' RACE was completed to as outlined by manufacturers’ protocols. To construct the recombinant plasmid, primers incorporating restriction sites were used to amplify the entire FhTLM ORF before cloning into a pET28a (Novagen) plasmid containing a 6X-His Tag as follows: Forward 5- GCGGCTAGCATGTGCAATTATGTGCCCGTTTTG-3 where the underlined sequence represents the NheI restriction site and Reverse 5- GCGCTCGAGTCAGATGACATTTGTTCCGGCAAG-3 where the underlined sequence represents the XhoI restriction site. The pET28aFhTLM plasmid was chemically transformed into *E. coli* Rosetta BL21 DE3/pLysS (Novagen, USA) and seeded onto LB agar supplemented with 30 μg/mL of kanamycin and 34 μg/mL of chloramphenicol. Positive clones were confirmed by colony PCR and individual sequencing. Thereafter bulk cultures were produced by inoculating 10 mL of LB with a single colony overnight and subsequently sub-culturing this into 500 mL of LB broth. Protein expression was induced by addition of IPTG at a final concentration of 1 mM, thereafter His-tagged FhTLM supernatant was purified over a Nickel resin column (Sigma-Aldrich). Before use recombinant protein was subject to phase separation to remove endotoxin residues [24]. Briefly, proteins were adjusted to 0.5 μg/mL concentration in a final volume of 5% Trition X-114. Samples were vortexed, incubated on ice for 5 min, incubated at 37 °C for 5 min and centrifuged at 5000 × *g* for 7 min. The lipid free fraction in the upper aqueous phase was retained and dialysed into PBS before further use.

### Functional assays

Unembryonated eggs released from fresh adult worms were collected and washed several times with dH_2_O. The egg suspension was adjusted to 1000 eggs/mL and 100 μL was distributed in 96-well plates. The eggs were then incubated with rFhTLM and human TGF-β (Peprotech) at 2.5 and 250 pg/mL containing penicillin (50 U/mL) and streptomycin (50 μg/mL) and gentamycin (10 μg/mL). The plate was covered with foil to prevent light activation and kept in a 30 °C dark incubator. At day 2, and 7 of incubation, the egg culture plate was removed and egg development was examined under an inverted microscope (Medline Scientific, UK). To assess egg production in adult worms, they were stimulated as above and eggs were counted in aliquots of culture supernatant under a stereo-microscope. Egg number was presented as eggs/gram of tissue wet weight for each fluke.

For an in vitro assessment of the rFhTLM effect on the newly excysted juveniles (NEJs) they were prepared as described elsewhere [25]. NEJs were cultured in RPMI-1640 containing 250 ng/mL of rFhTLM, or TGF-β at 37 °C with 5% CO_2_. Control worms were treated with PBS. After 24 h of cultivation, parasites were subsequently evaluated for viability and motility. Motility was observed under a stereo microscope and scored as +, ++, and +++. NEJs viability was determined by the MTT (3-[4, 5-dimethylthiazol-2-yl]-2, 5- diphenyltetrazolium bromide) assay according to manufacturers’ instructions (Sigma-Aldrich). Briefly, 100 μL of MTT (5 mg/mL) was added to cultures containing parasites and incubated for 30 min at 37 °C. The solution was then replaced with DMSO and incubated at RT for 1 h. Optical density (OD) was read at 540 nm of solutions in an ELISA plate after this.

### Lifestage PCR and in situ hybridisation

FhTLM gene expression was determined using cDNA prepared from various parasite life stages by conventional RT-PCR using *Taq* DNA Polymerase (Qiagen, UK) according to manufacturer’s instruction briefly, 5 μL of cDNA was used as template in a 50 μL PCR. FhTLM primers (5’-3’) were Forward GCTTGCCAATCGGGTGGACAGCAATTCA and Reverse CTGCATCCACATCCGAGAACAATGAG giving a band size of 342 bp. β-tubulin used as a housekeeping Forward GTATTGCATCGACAACGAAGCT and Reverse GTGCAAACGGGGGAACGG giving a band size of 192 bp.

To perform in situ hybridization the method of Pearson [26] was used in combination with a specific FhTLM DIG-labelled riboprobe (Roche UK). After fixing in 4% PFA specimens were moved to PBS with 0.5% Triton X-100 (PBSTx). Specimens were placed in 6% h2o2 followed by washing in 100% methanol; thereafter samples were rehydrated in a gradient of decreasing methanol. Specimens were incubated in PCR-grade proteinase K 10 μg/mL (Roche), re-fixed in 4% PFA and rinsed twice with PBSTx. Specimens were incubated in hybridization buffer (Sigma-Aldrich) for 2 h at 56 °C. This solution was refreshed and 1 μg/mL of hydrolysed DIG labelled probe was added and specimens incubated at 56 °C overnight. Thereafter specimens were washed with fresh hybridisation solution, saline sodium citrate buffer and maleic acid buffer with 0.1% Tween-20 (MABT). Specimens were then blocked in 1% Roche blocking solution and 20% horse serum for 2 h at RT and then incubated overnight with anti-DIG AP conjugated antibody at 4 °C overnight. Specimens were then washed in MABT 6 times for 20 min, colour was developed by the addition of NBT/BCIP substrate (Sigma-Aldrich), and PBSTx was used to stop signal development. Specimens were then cleared and mounted prior to examination. The sequences of the probes are as follows; sense - GCTTGCCAATCGGGTGGACAGCAATTCACATGTTGCACGCAATCGTTGAAAATTTACTTCTCCGAGATTGGGTGGGATCGTTGGATTATTCATCCGAAAAAATTCGAACCAAACTACTGCCGAGGATCCTGTCAAGTGAACGGTTTCCAGAGTACACACTACGAAGTGCTCAATCTTTTGTCACACAAAAATCTGACACAGCTGAAAGATGTGCCGCGTGGAACAATACAGTCTTGTTGTTATCCGACACGACGAA; anti-sense - CTGCATCCACATCCGAGAACAATGAGATTATGCAATGTGTGCATTCGCACGTCTTTATTTCGATCCAAATAGAGTAACGTGAACGTGGTTCGTCGTGTCGGATAACAACAAGACTGTATTGTTCCACGCGGCACATCTTTCAGCTGTGTCAGATTTTTGTGTGACAAAAGATTGAGCACTTCGTAGTGTGTACTCTGGAAACCGTTCACTTGACAGGATCCTCGGCAGTAGTTTGGTTCGAATTTTTTCGGATGAATAATCCAACGATCCCACCCAATCTCGGAGAAGTAAATTTTCAACGATTGCGTGCAACATGTGAATTGCTGTCCACCCGATTGGCAAGC.

### Statistical analysis

Statistical analysis was conducted using Prism 6.04 (Graphpad Software Inc.) details of the test used is indicated in figure legends along with *p*-values. A *p*-value of < 0.05 was taken to indicate significance.

## Results

### Identification of TGF-β superfamily members

To identify potential *F. hepatica* TGF-like sequences we screened for mammalian TGF-β-like sequences in the available *F. hepatica* Sanger EST database, however none were detectable. Conversely, TGF-β homologues were widespread in Platyhelminthes and nematode species. A PSI-Blast search using *Bos taurus* TGF-β1-3 identified 61 potential TGF-β homologues in nematodes and 11 in platyhelminths.

Identification of putative TGF-β superfamily members of *F. hepatica* was carried out by tBlastn searching of an unpublished genome database [19] using TGF-β protein sequences from closely related mammalian hosts of *F. hepatica* and the closely related helminth parasite, *S. mansoni*. The tBlastn search returned 4 hits for each of the *B. taurus* and *S. mansoni* queries; the top 3 hits originated from the same scaffolds numbered 40128, 30949 and 35064 (Additional file 1).

Sequence similarity was low between *F. hepatica* TGF-β-like candidates and mammalian TGF-β superfamily members. In contrast, the tBlastn query using SmInAct of *S. mansoni* identified mammalian homologs with high similarity particularly over approximately 100 amino acids of the C-terminal region.

This analysis showed at least 3 potential members of the TGF-β superfamily existed in the *F. hepatica* genome. To further confirm the presence of the hypothetical TGF-β superfamily, the translated peptide sequences retrieved from tBlastn were individually searched for the conserved TGF-β domain. The results showed that the three translated peptides were identified as possibly encoding proteins containing the TGF-β conserved domain. Subsequent Blastp searching against the bovine genome showed that scaffold 35064 (hereafter referred to as *Fasciola hepatica* TGF Like Molecule FhTLM) displayed 32% similarity to bovine inhibinβ chain (NP_001192912), a member of TGF-β subfamily. Furthermore, the deduced protein from scaffold 35064 was 59% similar to SmInAct of *S. mansoni* and inhibin-β B chain of *C. sinensis*. The remaining 2 scaffolds displayed most similarity to the members of BMP subfamily and were subsequently referred to as FhBMP1 FhBMP2.

### Phylogenetic analysis

**Figure 1:**
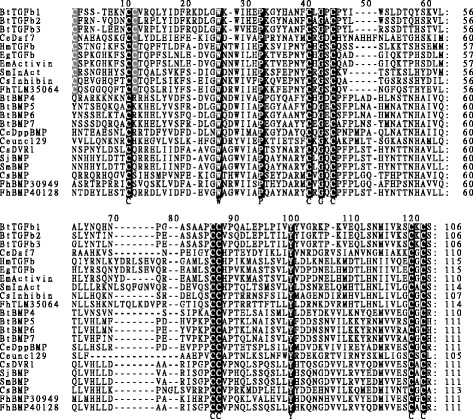
**Multiple sequence alignment of the deduced amino acid sequence at C-terminal region of the TGF-β superfamily members from** ***F. hepatica*** **and known TGF-β proteins.** Conserved residues among TGF-β superfamily are shaded in black background, additional cysteine residues conserved among TGF-β subfamily member are shaded in grey. Accession numbers for sequences used in this analysis are: *Bos taurus* TGF-β1 (BtTGFb1), NP_001159540; *B. taurus* TGF-β2 (BtTGFb2), DAA21445.1; *B. taurus* TGF-β3 (BtTGFb3), DAA25016; *B. taurus* BMP4 (BtBMP4), NP_001039342; *B. taurus* BMP5 (BtBMP5), DAA16713.1; *B. taurus* BMP6 (BtBMP6), DAA16077.1; *B. taurus* BMP7 (BtBMP7), DAA23024.1; *C. elegans* DAF-7 (CeDaf7), NP497265.1; *C. elegans* Dpp BMP (CeDppBMP), AAB04986.2; *C. elegans* unc129 (Ceunc129), NP501566.1; *C. sinensis* inhibin β B chain (CsInhibin), GAA54687.1; *C. sinensis* BMP (CsBMP), GAA53092.1; *C. sinensis* DVR1 (CsDVR1), GAA43145.2; *S. mansoni* InAct (SmInAct), XP_002577586.1; *S. mansoni* BMP (SmBMP), ABL74278.1; *S. japonicum* BMP (SjBMP), ADM48811.1*; H. microstoma* TGF-β family (HmTGFb), CDJ10022.1; *E. multilocularis* activin (EmActivin), CCV01195.1; *E. granulosus* TGF-β (EgTGFb), CDJ22078.1.

To examine sequence features of the candidate TGF-β superfamily members, we performed a multiple sequence alignment of known TGF-β proteins from both mammalian hosts and helminths. Bovine TGF-β1-3 and BMP4-7 were included as reference sequences for both protein subfamiles. The alignment indicated that all TGF-β superfamily members contain 7 conserved cysteine residues in their C-terminal region. Furthermore, mammalian and helminths TGF-β subfamily members contained an additional 2 cysteine residues. As expected, the putative FhTLM sequence contained 9 cysteine residues while FhBMP1 and FhBMP2 contained 7 cysteine residues. An alignment of protein sequences of the candidate *F. hepatica* TGF-β subfamily member and known TGF-β of other organisms is shown in Figure 1.

The evolutionary relationship between the putative TGF-β *F. hepatica* ligands and other members of TGF-β superfamily from helminths and mammalian hosts was investigated by multiple alignment and subsequent phylogenetic tree construction by PhyML software [27] using the WAG model [28]. Phylogenetic analysis of the conserved TGF-β domain was carried out on a total of 87 sequences of helminth and mammal TGF-β proteins. The phylogenetic tree displayed two major subfamilies of TGF-β members; one subfamily included 38 sequences of identified host and helminth TGF-β subfamily members. The second subfamily included 49 sequences from the BMP protein subfamily (Additional file 2).

Platyhelminth TGF-β homologues represented an individual phylogenetic clade. Inside this clade, three subclades of TGF-β homologues were clearly distinguished as belonging to free living flatworms, cestodes and trematodes. The trematode TGF-β subclade contained 2 branches; blood flukes and liver flukes. The FhTLM sequence was located in the liver fluke branch which showed greatest similarity to *C. sinensis* inhibin-β B chain.

### Cloning, gene structure, protein structure

**Figure 2:**
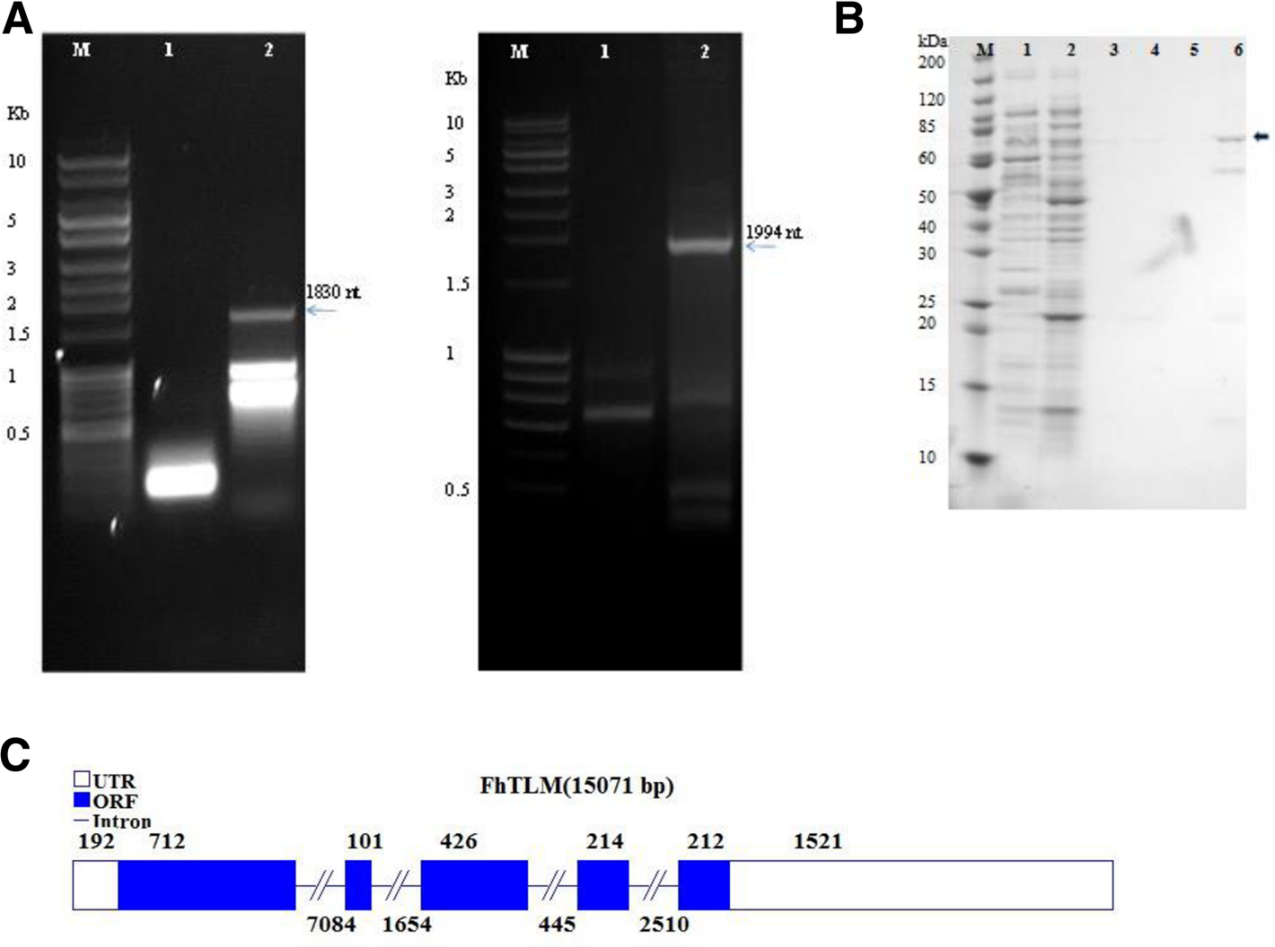
**Amplication, cloning and expression of FhTLM.** **(A)** 1.5% Agarose gel electrophoresis of amplified PCR fragments from 5’ RACE (left panel) and 3’ RACE (right panel). 5'-RACE product of 1830 bp and 3'-RACE product of 1994 bp were obtained from nested PCR, M, 1 Kb DNA ladder. **(B)** Coomassie stained SDS PAGE of protein expression from transformed Rosetta BL21 induced by 1 mM IPTG. Image represents native conditions. M; Protein marker, Lane 1; Supernatant uninduced Rosetta BL21, Lane2; Supernatant induced Rosetta BL21, Lane 3; Elution buffer 20 mM imidazole, Lane 4; Elution buffer 40 mM imidazole, Lane 5; Elution buffer 60 mM imidazole, Lane 6; Elution buffer 80 mM imidazole. **(C)** Organisation of the FhTLM gene depicting regions of the ORF (blue), exons (dashed lines), and UTRs (white) and their size in bases.

**Figure 3:**
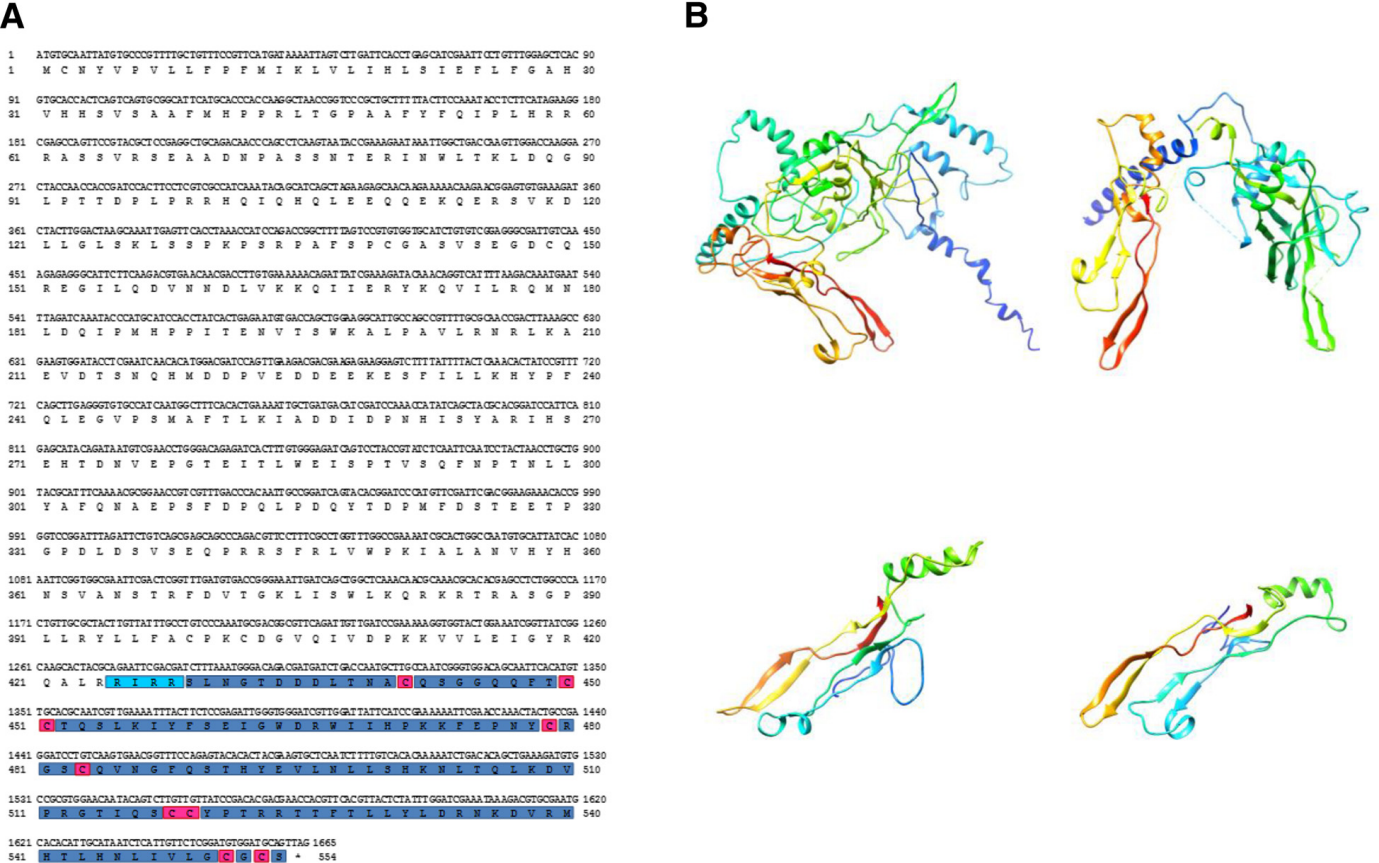
**Amino acid sequence and predicted 3D structure of FhTLM.** **(A)** Amino acid sequence showing corresponding nucleotide sequence along the putative cleavage site is shaded in light blue. The active TGF-β domain is shown blue shaded and sequences in pink background indicate the conserved cysteine residues in the active domain. **(B)** 3D structure of predicted FhTLM from Phyre2 by homology search modelling based on template 3rjrD (*Sus scrofa* TGF-β1). Full FhTLM protein; Full 3rjrD protein; FhTLM active domain; active domain of 3rjrD. On the models blue indicates the N-terminus and red indicates the C-terminus. The secondary structure components of FhTLM are composed of 13 α-helices and 16 β-sheets and 3rjrD are composed of 7 α-helices and 19 β-sheets.

The complete sequence of FhTLM was identified by means of rapid amplification of cDNA ends (RACE). Based on the partial sequence obtained from gene exonerate, primers were designed for amplifying the 5' and 3' ends of the FhTLM cDNA. 5' RACE amplification of the *F. hepatica* RACE cDNA produced 3 products ranging from 800 bp-1.9 kb (Figure 2A). A Blastn search of the sequenced 1860 bp PCR product against the *F. hepatica* genome database showed that the sequence of a 1860 bp fragment aligned with the sequence of *F. hepatica* genome scaffold 35064 representing the 5' end of FhTLM containing a 5'UTR and N-terminal region. The 3’ portion of FhTLM was amplified using primers specific to the conserved C-terminal domain of TGF-β. An amplicon of approximately 1994 bp (Figure 3A) was found to compromise the 3’ end of FhTLM, a 3’UTR and polyA tail. When sequenced this fragment mapped to scaffold 35064 of the *F. hepatica* genome. An overlapping sequence of approximately 420 bp between the 5' and 3' fragments displayed identical sequence. Manual compiling of these two cDNA ends resulted in a 3403 bp full-length sequence. The full length of FhTLM transcript was 3403 bp including a 192 bp 5’UTR, 1665 nt of coding region, and a 1547 nt of 3’ UTR including a poly A tail. The putative ORF of FhTLM ranges from the 193 nt to the 1857 nt bases. Recombinant FhTLM (rFhTLM) was generated by cloning the ORF sequence in pET28a at a NheI/XhoI site and transforming the vector into Rosetta blue cells. Purified rFhTLM appeared as a 65-70 kDa band on native SDS-PAGE gels (Figure 2B). A local Blastn search of FhTLM sequence against the *F. hepatica* genome allowed us to determine the structure of the gene encoding for FhTLM (Figure 2C).

The ORF encodes a deduced protein of 554 amino acid residues (Figure 3A), containing a conserved 126 amino acid long C-terminal domain. A furin proteolytic cleavage site, RIRR, was present at position 425–428 of the FhTLM molecule. Downstream of the cleavage site the C-terminal region contained 9 conserved cysteine residues characteristic of the TGF subfamily. No signal peptide sequences were detected. Within the active domain FhTLM is 60% identical (at the amino acid level) to *C. sinensis* inhibin-β B chain and 58% identical to SmInAct of *S. mansoni*. While, FhTLM is 35% similar to inhibin-β of human and cow and 32% similar to human and bovine TGF-β1 (Table 1).

**Table 1:** **Amino acid Sequence similarities between FhTLM and selected TGF-β homologues**

**Gene ID**	**Subject length**	**Max score**	**Query cover**	**E-value**	**Identity (%)**
TGF-β1 [*H. sapiens*]	116	52.8	99	9e^−15^	32
TGF-β1 [*B. taurus*]	116	52.8	99	9e^−15^	32
Inhibin beta B chain [*B. taurus*]	122	75.5	83	7e^−23^	35
Inhibin beta B chain [*H. sapiens*]	122	75.5	83	7e^−23^	35
DAF-7 [*C. elegans*]	120	72.0	87	2e^−21^	32
TGF-β1 family [*H. microstoma*]	132	120	99	4e^−40^	40
TGF-β1 family [*E. granulosus*]	135	130	100	9e^−44^	43
Activin [*E. multilocularis*]	135	130	100	9e^−44^	43
Inhibin beta B chain [*C. sinensis*]	130	179	99	63e^−63^	60
SmInAct [*S. mansoni*]	130	174	99	42e^−61^	58

Protein models of FhTLM were constructed by searching against the Protein Data Bank (PDB) on Phyre2 [29]. The results showed that predicted FhTLM models of the propeptide and conserved active domain share fold structure with *Sus scrofa* TGF-β1 PDB crystal structure (c3rjrD). The predicted structure consists of 13 α-helices and 16 β-sheets present in the secondary structure of FhTLM (Figure 3B). The final model for FhTLM precursor was built from 65% coverage sequence based on the c3rjrD template corresponding to the sequence at the C-terminal with >90% confidence. The predicted model of active domain sequence was constructed from 87% of the FhTLM sequence (109 amino acids) and demonstrated 2 α-helices and 7 β-sheets. The confidence score of this model was 100% indicating that the model was correct and the FhTLM and TGF-β1 are truly homologous.

### Lifestage expression and localisation

**Figure 4:**
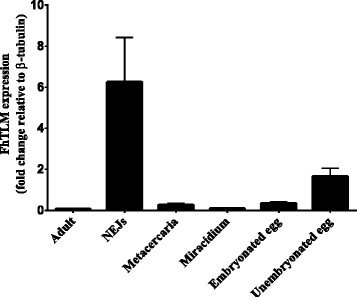
**Life stage PCR detection of FhTLM transcript by PCR using cDNA template form the indicated stages of** ***F. hepatica*** **.** The FhTLM product is 342 bp while β-tubulin is 196 bp. Relative quantification (fold change) of FhTLM mRNA expression compared to the corresponding of the β-tubulin control from the identical developmental stage of *F. hepatica* including adult, NEJ, metaceracaria, miracidium, embryonated egg and unembryonated egg. Values were analyzed from three independent PCRs, with similar results and values shown are representative means ± SD from a single experiment.

Semi-quantitative PCR analysis was used to assess the expression of FhTLM mRNA throughout liver fluke development. Gene specific primers were designed against regions flanking introns in FhTLM mRNA using β-tubulin as a house-keeping gene. The predicted size of PCR products for FhTLM and β-tubulin are 342 and 192 bp, respectively. FhTLM and β-tubulin transcripts were detected at all parasite stages examined. However, FhTLM and β-tubulin mRNA were expressed at varying levels within different lifestages. Using ImageJ software, values for FhTLM expression were normalized to β-tubulin expression levels. Relative quantification revealed that highest levels of FhTLM were detected in NEJ and unembryonated eggs whereas adult and miracidial stages displayed the lowest levels of expression (Figure 4).

**Figure 5:**
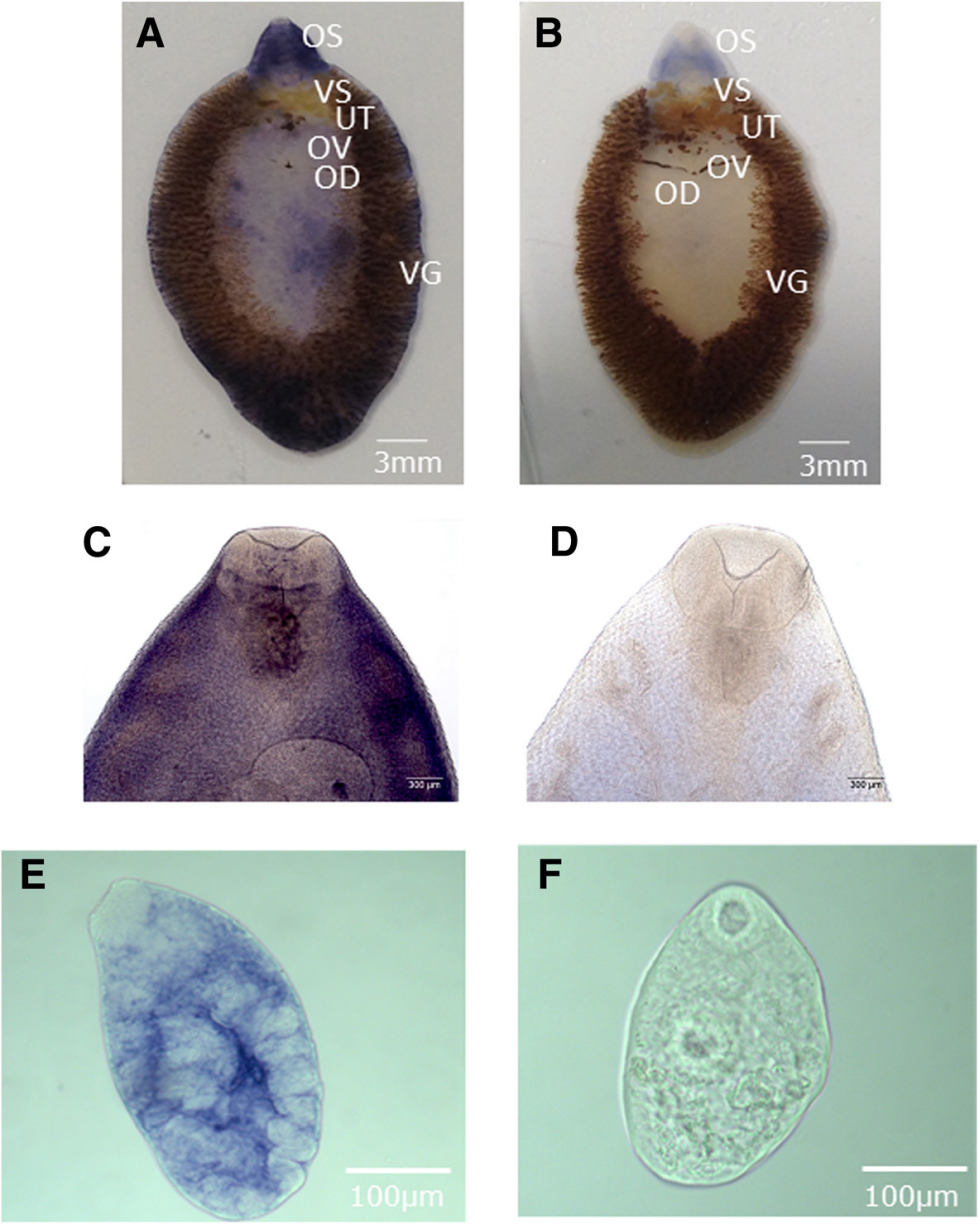
**Whole mount in situ hybridization of FhTLM in adult liver fluke.** **(A)** Adult stage treated with antisense FhTLM probe showing a blue precipitate at several parts of the worm cephalic cone including oral sucker (OR), ventral sucker (VS), no staining detected in uterus (UT), oviduct (OV) and vitelline gland (VG). **(B)** Sense control showing unstained worm. Scale bar, **A** & **B**, indicates 3 mm. Localization of FhTLM transcript on the cephalic cone of the adult worms **(C)**. Control worm with no visible signal **(D)**. Scale bar, **C** & **D**, indicates 300 μm. NEJ stained showing extensive straining except in the pharynx, oral and ventral suckers **(E)**. **(F)** control NEJs. Scale bar indicates 100 μm.

We next conducted in situ hybridization to confirm our PCR results above and establish the location of the mRNA within parasite tissues. In adult specimens we detected the transcript widely spread on the tegument and sub-tegumental region of the adult fluke (Figure 5A). A strong signal was present in the anterior and posterior end (Figure 5A). A faint staining pattern along the lateral part of worm starting from shoulder to tail was also seen. A positive reaction could also be observed as a fine dot pattern spread over the ventral surface of the treated flukes (Figure 5C). In situ hybridization of adult liver fluke showed no signal within the reproductive organs. In NEJs (Figure 5E), the FhTLM transcript was widely dispersed throughout the internal structures with an intense signal around the centre of the body. No signal was present within the pharynx, or the anterior and posterior ends of NEJs. The overall intensity of in situ hybridisation staining within NEJs confirmed the expression analysis above. Sparse staining was seen in metacercariae, eggs, and miracidium.

### Intrinsic effects of FhTLM in ***F. hepatica***

**Figure 6:**
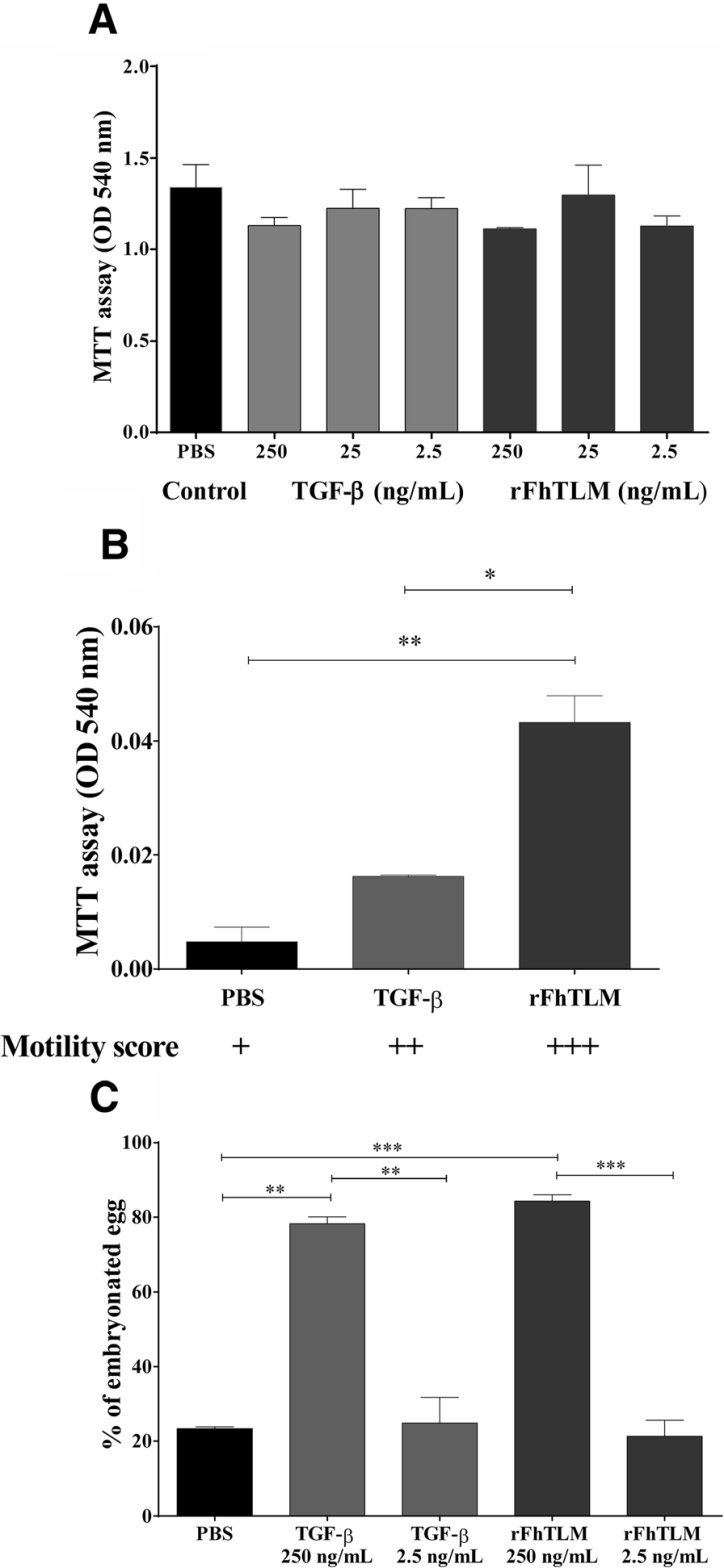
**Intrinsic biological effects of FhTLM are lifestage specific.** **(A)** Effect of rFhTLM on the viability of adult liver flukes. Viability was determined by MTT reduction after of 24 hours of incubation. Results are plotted as mean ± SEM of triplicate readings. from one of two independent experiments. **(B)** Effects of rFhTLM on the motility and viability of NEJs. Viability of the NEJs was determined by MTT reduction at 24 h of incubation 250 ng/mL of rFhTLM and TGF-β, or PBS. Results are plotted as the mean OD reading values ± SEM from three replicates from one of two independent experiments. Motility was recorded in the same experiments is indicated underneath using a semi-quantitative scale. **(C)** Biological effect of rFhTLM on egg embryonation. Unembryonated eggs were incubated with rFhTLM and TGF-β at the indicated dose, or dH2O for 7 days and the egg development was examined microscopically. Results are represented as mean ± SEM of % embryonated egg. Data in panel **B** is analysed by 1-way anova and data in panel **C** is analysed using 2-way anova. Significant differences are *P < 0.05, **P < 0.01 and ***P < 0.001. All results are representative of one of two independent experiments.

The effect of rFhTLM on adult liver flukes was tested by incubating specimens with recombinant protein and conducting observations after 24 h. Measurements of viability (Figure 6A), via MTT assay, and egg production (Table 2) showed no significant differences in the viability of flukes and number of eggs detected between specimens from all treatments. As controls, adult worms were also exposed to mammalian TGF-β with similarly negative results.

**Table 2:** **Biological effects of rFhTLM on egg production of the adult liver fluke – displayed as average egg no./gram of fluke weight**

**Treatment (ng/mL)**		**Average egg no./g**
*PBS*		40332.92
*TGF-β*	*250*	15626.23
	*25*	14851.90
	*2.5*	27917.94
*rFhTLM*	*250*	14790.10
	*25*	16414.05
	*2.5*	10833.33

Similar experiments with NEJs producing strikingly contrasting results, all NEJs remained viable after 24 h. Their motility was examined by observation and assigned semi-quantitative scores. NEJs treated with rFhTLM were the most active (i.e. exhibited most movement) compared to those observed that were treated with TGF-β, however both of these groups contained parasites with greater motility when compared to PBS treated NEJs (Figure 6B). This data would appear to correlate with our MTT results, as TGF-β treated NEJs displayed enhanced viability compared to PBS treated NEJs but this was still lower than the group treated with rFhTLM who displayed the greatest levels of viability (Figure 6C).

We next sought to test the effects of rFhTLM on egg development as we had noticed a peak in expression in unembryonated eggs. Unembryonated eggs can be cultured in vitro to develop the egg mass to an embroynated state and hatch after 7–9 days. Batches of eggs were incubated with rFhTLM, TGF-β, or control for 7 days before examination. A significant difference in egg development was observed between treated and non-treated groups. rFhTLM and TGF-β1 showed similar effects in that they increased embryonation of eggs and development of the egg mass (Figure 6C). There was slightly higher percentage of embryonation induced by rFhTLM when compared to TGF-β. In control groups only 25% of egg showed signs of development while eggs treated with 250 ng/mL of TGF-β or rFhTLM had 78.21% and 84.30% of eggs developed, respectively. At a low concentration of both TGF-β and rFhTLM development levels were similar to control group with no statistical differences detected.

## Discussion

In this study, we have identified mammalian TGF-β homologues from the *F. hepatica* genome database using a bioinformatics approach. Our analysis suggests that there are three TGF-β superfamily members present in the *F. hepatica* genome. Upon further characterisation we found that two of these correspond to BMP homologues and one is a TGF-β homologue which we termed FhTLM. A full sequence of the cDNA encoding this protein was isolated using RACE. The putative FhTLM contains a prodomain and conserved TGF-β protein domain without a signal peptide. Similar to other TGF-β subfamily members, FhTLM contains a highly conserved region in the C-terminal with 9 cysteine residues which are involved in the formation of the disulfide bridges in the cysteine knot within and between chains, a hallmark of the TGF-β protein subfamily.

We found that the cDNA sequence displays a very low identity when Blasted against available datasets, the top hit by Blastn was found to be the *S. mansoni* SmInAct mRNA sequence. At the protein level, the deduced FhTLM sequence when compared to other mammalian TGF-β proteins, displayed low similarity. The similarity between the FhTLM C-terminal and that of a nematode, *C. elegans*, and mammalian TGF-β, bovine and human, are between 32-35%. However, FhTLM exhibits a high degree of conservation to TGF-β homologues from trematodes and cestodes. Sequence analysis revealed over 50% similarity between the conserved domains of TGF-β members of the plathyhelminths. The amino acid sequence of FhTLM shares 60% identity with *C. sinensis* inhibin-β B chain and 58% identity to *S. mansoni* SmInAct.

Phylogenetic analysis of the TGF-β members demonstrated that FhTLM clustered within the platyhelminth group consisting of TGF-β homologues from the subclasses Turbellaria, Cestoda and Trematoda. Our phylogenetic tree was reproducible confirming the described relationship between free living worms and parasitic worms. The parasitic flatworm TGF-βs are clustered in the same group, separate from free living planaria inferring that the TGF-β of the parasitic worms share common function (s) which might be specific and vital to establishing a parasitic life cycle. To date only 5 TGF-β homologous ligands have been reported in parasitic flatworms including *S. mansoni* [15], *C. sinensis* [30], *E. multilocularis* [31], *E. granulosus* and *H. microstoma* [32]. We observed that all parasitic flatworms including *F. hepatica* contain only one TGF-β homologue but vary in the number of BMP homologues whereas free living planaria have 2 TGF-β like proteins [33].

The major conserved developmental signalling pathways including Wingless related (Wnt), Delta/Notch, Hedgehog (Hh) and TGF-β have been identified in all bilateral animals studied. It is very clear that the components of these signalling pathways are evolutionary conserved amongst animal species in terms of structure and function [2]. TGF-β super family proteins (particularly Nodal/Activin and BMPs) are essential during early embryogenesis and developmental processes. These pathways operate continuously in fully developed animals playing important roles in tissue morphogenesis and homeostasis [3]. Defects in TGF-β signalling pathways can result in remarkable changes in the phenotype of multiple model animals [7,34] and lead to serious clinical diseases in humans [3,5]. Herein we show a conservation of these processes within the *F. hepatica* TGF family member, FhTLM. Our results indicate that rFhTLM can increase the process of embryonation within eggs and increase the viability and motility of NEJs. Interestingly our results demonstrated that there was no effect of rFhTLM on adult worms. This stage restricted mode of action also aligns with the results we obtained when investigating the pattern of FhTLM expression within different lifestages, where NEJs demonstrated the greatest levels of expression and levels within adults were relatively low. The next highest levels of expression after those seen in NEJs were seen in unembryonated eggs which would confirm that the actions of FhTLM seen here in our experiments could arise from endogenous protein. Most likely, in our in vitro experiments using recombinant FhTLM and human TGF-β these proteins were actively scavenged from the environment. Our previous work has shown that eggs interact with their surroundings [35]. Our results are in line with the reported function of SmInAct which is also involved in embryonation of eggs in *S. mansoni* [15]. However contrary to findings in *S. mansoni* we did not find FhTLM to be highly expressed within the reproductive organs of adult worms, this may be due to *F. hepatica* being hermaphrodite in nature or a more species-intrinsic function of SmInAct.

During our initial screening for a TGF-like molecule within *F. hepatica* antigens we demonstrated the ability of native and recombinant FhTLM to bind the mammalian receptor complex and initiate signalling. This is interesting given the reported cross-talk between parasite and host ligands for TGF-like molecules in other parasite species *H. polygyrus* [36] and *B. malayi* [37]. Whether this cross-talk occurs in the case of *F. hepatica* infection remains to be investigated, but could yet yield further control targets.

In conclusion we have identified for the first time members of the *F. hepatica* TGF protein superfamily. Cloning and further characterisation of a TGF-β/activin like member of this family, FhTLM, revealed a distinct expression pattern. The biological effects of a recombinant form of this protein demonstrated activities that were limited to enhanced embryonation and survival of juvenile parasites. Further analysis using RNAi will determine the full phenotype of FhTLM and the function of other TGF superfamily members from *F. hepatica*. FhTLM may yet be a promising diagnostic target for the identification of animals harbouring pre-patent infections due to the high, and specific, levels of expression of FhTLM seen in NEJs. Likewise characterisation of the function of FhTLM would indicate that it is essential to NEJ survival and motility, and thus possibly peritoneal migration making it a target in terms of a vaccine designed to halt NEJ migration and establishment.

## Additional files

Additional file 1:
**BLASTn output from genome wide analysis of** ***F. hepatica.*** tBlastn output window from genome wide analysis using SmInAct as a query sequence, displaying matching to scaffolds 35064, 40128, 30949. These scaffolds contained the putative FhTLM sequence. Additional file 2:
**Phylogenetic relationships of mammalian TGF-β and helminth TGF-β homologues.** The phylogenetic tree represents the relationship of the representative sequences of mammalian TGF-β family members and TGF-β homologues of helminths. Numbers at nodes are boostrap value based on 1000 bootstrap replicates.

## References

[1] Huminiecki L, Goldovsky L, Freilich S, Moustakas A, Ouzounis C, Heldin CH (2009). Emergence, development and diversification of the TGF-βsignalling pathway within the animal kingdom. BMC Evol Biol.

[2] Richards GS, Degnan BM (2009). The dawn of developmental signaling in the metazoa. Cold Spring Harb Symp Quant Biol.

[3] Wu MY, Hill CS (2009). TGF-β superfamily signaling in embryonic development and homeostasis. Dev Cell.

[4] Kusserow A, Pang K, Sturm C, Hrouda M, Lentfer J, Schmidt HA, Technau U, von Haeseler A, Hobmayer B, Martindale MQ, Holstein TW (2005). Unexpected complexity of the Wnt gene family in a sea anemone. Nature.

[5] ten Dijke P, Arthur HM (2007). Extracellular control of TGF-β signalling in vascular development and disease. Nat Rev Mol Cell Biol.

[6] Zhang X, Zhang Y (2012). DBL-1, a TGF-β, is essential for *Caenorhabditis elegans* aversive olfactory learning. Proc Natl Acad Sci U S A.

[7] Morita K, Chow KL, Ueno N (1999). Regulation of body length and male tail ray pattern formation of *Caenorhabditis elegans* by a member of TGF-β family. Development.

[8] Gumienny TL, Savage-Dunn C (2013) TGF-β signaling in *C. elegans.* In: The *C. elegans* Research Community (eds) WormBook doi/10.1895/wormbook.1.22.2.

[9] Viney ME, Thompson FJ, Crook M (2005). TGF-β and the evolution of nematode parasitism. Int J Parasitol.

[10] Brand AM, Varghese G, Majewski W, Hawdon JM (2005). Identification of a DAF-7 ortholog from the hookworm *Ancylostoma caninum*. Int J Parasitol.

[11] Freitas TC, Arasu P (2005). Cloning and characterisation of genes encoding two transforming growth factor-beta-like ligands from the hookworm, Ancylostoma caninum. Int J Parasitol.

[12] Crook M, Thompson FJ, Grant WN, Viney ME (2005). daf-7 and the development of *Strongyloides ratti* and *Parastrongyloides trichosuri*. Mol Biochem Parasitol.

[13] Massey HC, Castelletto ML, Bhopale VM, Schad GA, Lok JB (2005). Sst-tgh-1 from Strongyloides stercoralis encodes a proposed ortholog of daf-7 in *Caenorhabditis elegans*. Mol Biochem Parasitol.

[14] McSorley HJ, Grainger JR, Harcus Y, Murray J, Nisbet AJ, Knox DP, Maizels RM (2010). daf-7-related TGF-β homologues from Trichostrongyloid nematodes show contrasting life-cycle expression patterns. Parasitology.

[15] Freitas TC, Jung E, Pearce EJ (2007). TGF-β signaling controls embryo development in the parasitic flatworm *Schistosoma mansoni*. PLoS Pathog.

[16] Freitas TC, Pearce EJ (2010). Growth factors and chemotactic factors from parasitic helminths: molecular evidence for roles in host-parasite interactions versus parasite development. Int J Parasitol.

[17] Liu R, Zhao QP, Ye Q, Xiong T, Tang CL, Dong HF, Jiang MS (2013). Cloning and characterization of a bone morphogenetic protein homologue of *Schistosoma japonicum*. Exp Parasitol.

[18] Hewitson JP, Grainger JR, Maizels RM (2009). Helminth immunoregulation: the role of parasite secreted proteins in modulating host immunity. Mol Biochem Parasitol.

[19] Hodgkinson J, Cwiklinski K, Beesley NJ, Paterson S, Williams DJ (2013). Identification of putative markers of triclabendazole resistance by a genome-wide analysis of genetically recombinant *Fasciola hepatica*. Parasitology.

[20] Predication of Genes based on Exonerate. https://github.com/ADAC-UoN/predict.genes.by.exonerate.pipeline. Accessed on 14 March 2013

[21] Edgar RC (2004). MUSCLE: multiple sequence alignment with high accuracy and high throughput. Nucleic Acids Res.

[22] Galtier N, Gouy M, Gautier C (1996). SEAVIEW and PHYLO_WIN: two graphic tools for sequence alignment and molecular phylogeny. Comput Appl Biosci.

[23] Gouy M, Guindon S, Gascuel O (2010). SeaView version 4: A multiplatform graphical user interface for sequence alignment and phylogenetic tree building. Mol Biol Evol.

[24] Aida Y, Pabst MJ (1990). Removal of endotoxin from protein solutions by phase separation using Triton X-114. J Immunol Methods.

[25] Wilson LR, Good RT, Panaccio M, Wijffels GL, Sandeman RM, Spithill TW (1998). *Fasciola hepatica*: characterization and cloning of the major cathepsin B protease secreted by newly excysted juvenile liver fluke. Exp Parasitol.

[26] Pearson BJ, Eisenhoffer GT, Gurley KA, Rink JC, Miller DE, Sanchez Alvarado A (2009). Formaldehyde-based whole-mount *in situ* hybridization method for planarians. Dev Dyn.

[27] Guindon S, Dufayard JF, Lefort V, Anisimova M, Hordijk W, Gascuel O (2010). New algorithms and methods to estimate maximum-likelihood phylogenies: assessing the performance of PhyML 3.0. Syst Biol.

[28] Whelan S, Goldman N (2001). A general empirical model of protein evolution derived from multiple protein families using a maximum-likelihood approach. Mol Biol Evol.

[29] Kelley LA, Sternberg MJ (2009). Protein structure prediction on the Web: a case study using the Phyre server. Nat Protoc.

[30] Wang X, Chen W, Huang Y, Sun J, Men J, Liu H, Luo F, Guo L, Lv X, Deng C, Zhou C, Fan Y, Li X, Huang L, Hu Y, Liang C, Hu X, Xu J, Yu X (2011). The draft genome of the carcinogenic human liver fluke *Clonorchis sinensis*. Genome Biol.

[31] Brehm K (2010). *Echinococcus multilocularis* as an experimental model in stem cell research and molecular host-parasite interaction. Parasitology.

[32] Tsai IJ, Zarowiecki M, Holroyd N, Garciarrubio A, Sanchez-Flores A, Brooks KL, Tracey A, Bobes RJ, Fragoso G, Sciutto E, Aslett M, Beasley H, Bennett HM, Cai J, Camicia F, Clark R, Cucher M, De Silva N, Day TA, Deplazes P, Estrada K, Fernandez C, Holland PW, Hou J, Hu S, Huckvale T, Hung SS, Kamenetzky L, Keane JA, Kiss F (2013). The genomes of four tapeworm species reveal adaptations to parasitism. Nature.

[33] Gavino MA, Wenemoser D, Wang IE, Reddien PW (2013). Tissue absence initiates regeneration through Follistatin-mediated inhibition of Activin signaling. Elife.

[34] Lehmann P, Rank P, Hallfeldt KL, Krebs B, Gartner R (2006). Dose-related influence of sodium selenite on apoptosis in human thyroid follicles in vitro induced by iodine, EGF, TGF-β, and H_2_O_2_. Biol Trace Elem Res.

[35] Moxon JV, Flynn RJ, Golden O, Hamilton JV, Mulcahy G, Brophy PM (2010). Immune responses directed at egg proteins during experimental infection with the liver fluke *Fasciola hepatica*. Parasite Immunol.

[36] Grainger JR, Smith KA, Hewitson JP, McSorley HJ, Harcus Y, Filbey KJ, Finney CA, Greenwood EJ, Knox DP, Wilson MS, Belkaid Y, Rudensky AY, Maizels RM (2010). Helminth secretions induce *de novo* T cell Foxp3 expression and regulatory function through the TGF-β pathway. J Exp Med.

[37] Gomez-Escobar N, Gregory WF, Maizels RM (2000). Identification of tgh-2, a filarial nematode homolog of *Caenorhabditis elegans* daf-7 and human transforming growth factor beta, expressed in microfilarial and adult stages of *Brugia malayi*. Infect Immun.

