# Supplementary figures and images for: TGF-β superfamily members from the helminth Fasciola hepatica show intrinsic effects on viability and development
Source: Vet Res. 2015 Mar 11;46:29. doi: 10.1186/s13567-015-0167-2 (PMC4354977; doi:10.1186/s13567-015-0167-2)

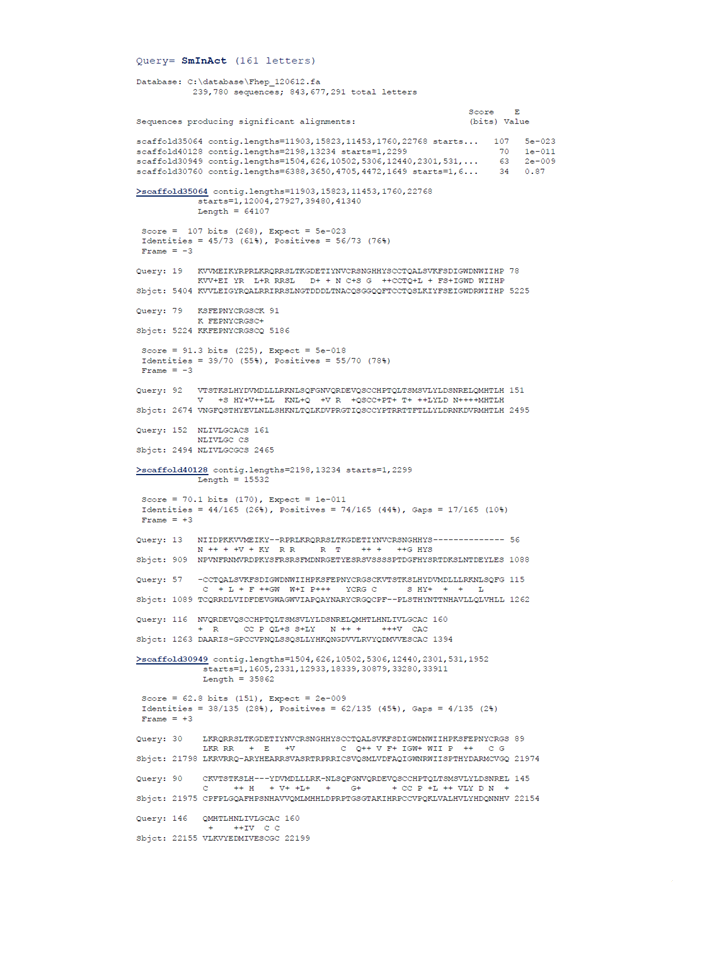

Supplement: Additional file 1: — BLASTn output from genome wide analysis of F. hepatica. tBlastn output window from genome wide analysis using SmInAct as a query sequence, displaying matching to scaffolds 35064, 40128, 30949. These scaffolds contained the putative FhTLM sequence. [file 13567_2015_167_MOESM1_ESM.tif]

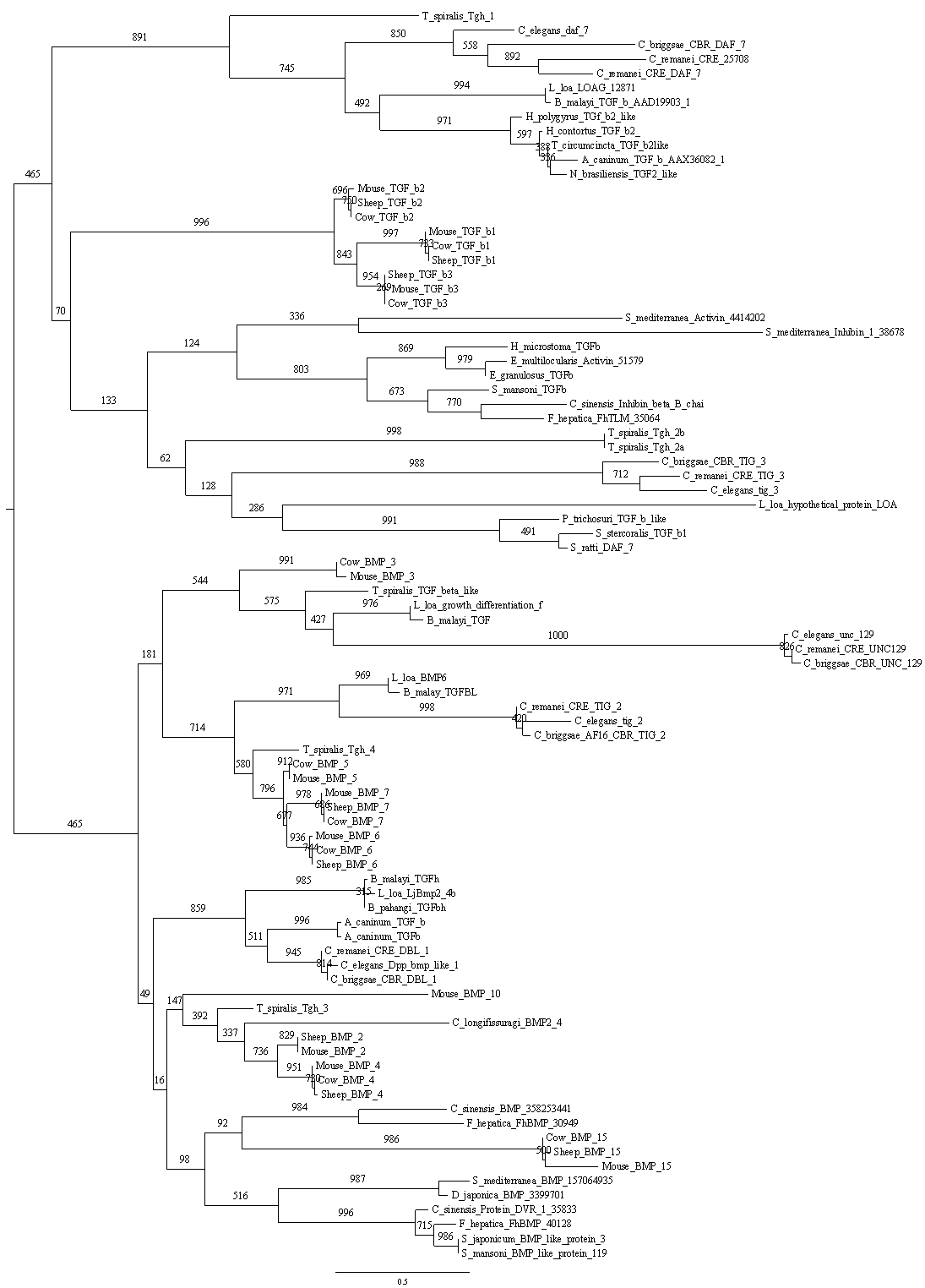

Supplement: Additional file 2: — Phylogenetic relationships of mammalian TGF-β and helminth TGF-β homologues. The phylogenetic tree represents the relationship of the representative sequences of mammalian TGF-β family members and TGF-β homologues of helminths. Numbers at nodes are boostrap value based on 1000 bootstrap replicates. [file 13567_2015_167_MOESM2_ESM.tif]
